# Validation of digital photographs, as a tool in 24-h recall, for the improvement of dietary assessment among rural populations in developing countries

**DOI:** 10.1186/1475-2891-11-61

**Published:** 2012-08-29

**Authors:** Claudia E Lazarte, Ma Eugenia Encinas, Claudia Alegre, Yvonne Granfeldt

**Affiliations:** 1 Department of Food Technology, Lund University, P.O. Box 124, Lund 221 00, Sweden; 2 Food and Natural Products Center, San Simon University, Cochabamba, Bolivia; 3 Departament of Nutrition, San Simon University, Cochabamba, Bolivia

**Keywords:** Weighed record, 24-h recall, Digital photographs, Photo atlas, Developing countries

## Abstract

**Background:**

Improvement of traditional methods for dietary assessment is necessary, especially in rural areas where it is more difficult to succeed with self-reporting methods. This study presents and validates a method for improving accuracy when measuring food and nutrient intake of individuals in rural areas. It is called the “Food photography 24-h recall method” (FP 24-hR) and is a modified 24-h recall with the addition of a digital food photography record and a photo atlas.

**Methods:**

The study was carried out in a rural area in the tropical region of Bolivia; 45 women participated. Validation of the method was made by comparing it with a reference method, the Weighed Food Record (WFR). During the FP 24-hR, digital photographs were taken by the subjects of all food consumed during a day and a 24-h recall questionnaire was conducted by an interviewer. An estimate of the amount of food consumed was made using a photo atlas and the photographs taken by the subjects. For validation, comparison was made between the calculations, by both methods, of the levels of food, and nutrient, intake.

**Results:**

The comparison was made in 10 food categories; most of which were somewhat underestimated from −2.3% (cassava) to −6.8% (rice), except for beverages (+1.6%) and leafy vegetables (+8.7%), which were overestimated. Spearman’s correlation coefficients were highly significant (*r* from 0.75 for eggs to 0.98 for potato and cassava). Nutrient intakes calculated with data from both methods showed small differences from -0.90% (vitamin C) to -5.98% (fat). Although all nutrients were somewhat underestimated, Pearson^′^s coefficients are high (>0.93 for all) and statistically significant. Bland Altman analysis showed that differences between both methods were random and did not exhibit any systematic bias over levels of food and nutrient intake, with acceptable 95% limits of agreement.

**Conclusion:**

The FP 24-hR exhibits acceptable differences when compared with a WFR, digital photos are useful as a memory aid for the subjects during 24-h recall and as an estimation tool. The method is suitable for assessing dietary intake among rural populations in developing countries.

## Background

Nutritional assessment in many low-income countries emphasizes new simple, non-invasive approaches that can be used to measure the risk of both nutrient shortages and excesses, as well as to monitor and evaluate the effects of a nutrition intervention. One approach is to use the dietary assessment methods which can identify any nutritional deficiencies by measuring the food consumption of individuals [1].

In some rural populations in low-income countries a weighed food record, completed by trained research assistants in the households, has been used as the most precise method available for estimating the usual food and nutrient intake of individuals, because some subjects are not literate or cannot use the scales [2,3], in Bolivia, the illiteracy in rural areas is 37.9% for women with 15 years or more, and 15.7% for men [4]. However, the method is time-consuming and expensive and the usual eating pattern of the respondents can easily be disrupted. Therefore the 24-h recall is being used widely to assess the dietary intake of individuals [5-7]; the method is quick and economical, it can be used equally well with both literate and illiterate subjects, and the respondent burden is small. Nevertheless, the success of the method depends on the subject’s memory, the ability of the subject to conduct accurate estimates of portion sizes consumed, and the persistence of the interviewer [1]. Furthermore, it has been reported that the 24-h recall applied as the sole method in rural populations resulted in a systematic negative bias that lead to significant underestimates of average daily energy and nutrient intake compared with that obtained by the weighed record [8] as well as the misreporting of energy and micronutrient intake [9].

All methods used to assess self-reported daily dietary intake have several limitations in terms of the accuracy of the portion size estimation [1,10]. To improve the accuracy of dietary assessment methods and overcome their limitations it is recommended to make the existing techniques more sensitive to community specifics by using multiple measurement methods [3], as there is a large variation from community to community with respect to staple foods, their preparation and dietary habits in general. One of the main errors to occur in the measurement of food consumption in dietary surveys is the assessment of portion sizes; therefore standard portions, household measures, food models and pictures are used as aids for the quantitative estimation of food in dietary data collection [11]. Food photographs depicted in standardized portion sizes (small, medium and large portions which are meant to be representative of the range of portion sizes actually consumed), organized in a booklet or atlas have been shown to be helpful in improving the accuracy of food quantification [12-15].

As a new approach the inclusion of digital photographs has been used to estimate portion size by taking photos of food and meals before and after consumption and by making food estimations either with the digital photographs alone or by comparing them with standard photographs. This method was validated mostly by comparing it with weighed records (as a reference method). Studies have been conducted in a variety of settings such as schools, colleges, university cafeterias [16-18], laboratories [19,20], hospitals or community centers [21,22], and in free-living conditions [20,23,24]. The results indicate that digital photographs are useful for assessing dietary intake in individuals, and for reducing the respondent burden associated with completing food records. To our knowledge, the use of digital photographs has not yet been validated or used in rural populations in low-income countries.

The aim of the present study was to develop and validate a modified 24-h recall method with digital food photographs as a tool for subjects to recall their intake, and a photo atlas with standard portion sizes of the foods commonly consumed in the area to simplify the estimation of consumed portions. The validity of the method was assessed by comparing the results with a reference method WFR running in parallel. The modifications were made to adapt the food photographs for use among rural populations in low-income countries where there may be a limited ability to read or write. The method developed is designed to be used in a further study for assessing the dietary intake of patients with leishmaniasis in the same area.

## Methods

### Subjects and study design

Women aged 20–52 years, from a rural area named Eterazama, a tropical region located 180 km east of Cochabamba-Bolivia, participated. A nurse from the local health center visited women in their homes within a 0.5 and 3 km radius around the health center and invited them to participate. Their participation depended on their willingness to be followed closely for one day during the preparation and consumption of their meals.

**Figure 1:**
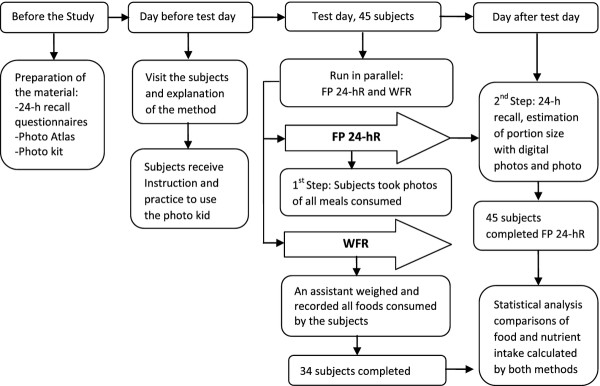
Design of the study: Validation of the developed method FP 24-hR by comparing it with WFR.

Figure 1 shows the design of the study. A modified 24-h recall method in 2 steps, so-called FP 24-hR was developed. In the first step, digital photographs are taken by the subjects, of the foods they consume over a 24 hour period; in the second step, one day after, during an interview following a 24-h recall questionnaire, the subjects estimate and report the quantities of food consumed the day before. Their digital photographs help them to recall all foods and also to estimate the portion size by comparing them with standard food photographs in a photo atlas. The FP 24-hR was validated with a reference method, WFR, in which weighed amounts of the food consumed were recorded by assistants in the subject^′^s home. The two methods were run in parallel during a test day.

The Ethics Committee of the Faculty of Medicine at Lund University approved the study.

### 24-h recall questionnaire

A 24-h recall questionnaire was elaborated according to guidelines given in [Gibson (2005]), and pre-tested with respondents in the area in question in order to ensure that the questions were relevant and understandable. The questionnaire has questions about the name of the foods and meals consumed, whether food intake was normal that day, and if there was any consumption of medicines or vitamin-mineral supplements; also, place and time of consumption are listed for: breakfast, mid-morning snack, lunch, mid-afternoon snack and dinner.

### Photo atlas

A photo atlas with color photographs of 78 common foods consumed in the area, in various portion sizes, was included to assist the interviewer and participants in estimating the sizes of the portions. A total of 334 photos, divided into 8 food groups, that is meat, cereals, legumes, tubers, vegetables, fruits, composite meals and drinks, are depicted in the atlas.

To prepare the photo atlas, we used population-based data as suggested by Nelson and Haraldsdóttir (1998). Nutritionists visited families in the area of intervention to acquire some knowledge of the most commonly consumed foods, the portion sizes and the tableware used. This information was collected in an open questionnaire and used to design the album in terms of the number of items, the number of portion sizes and the kind of plates on which the food should be photographed.

The photographs of the food in the photo atlas were taken at approximately the same angle of 90° and distance of 50 cm, above the plate. A second photograph, with an approximately 45° angle, used to show differences between portion sizes depending on the height of the food on a flat plate and depth in a soup plate, was taken when necessary. The plates were placed on a table mat with 1.5 cm grids marked out. It was deemed useful to keep a standard background for the photographs. Additionally, reference objects of a spoon, fork or knife were placed next to the dish to provide some idea of scale of the dish size.

**Figure 2:**
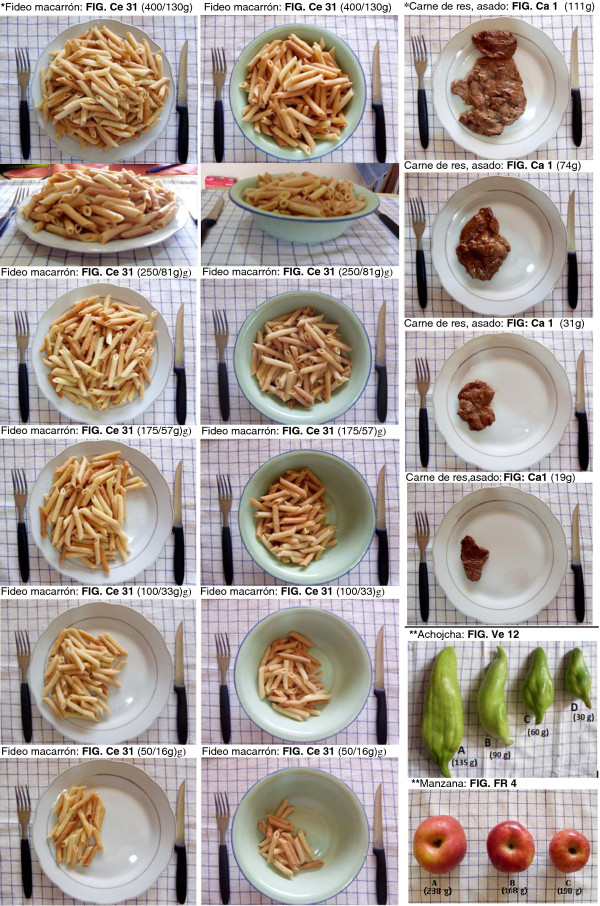
Example of photographs from the photo atlas shows portion sizes of cooked and raw food. *Example of cooked food in different dishes and from different angles: the name of the food (in Spanish) and weight of the portion is shown on the top of each photograph. In cases where there is a change in the weight during cooking, both weights are shown (weight of cooked food/weight of raw food). **Example of raw food, the weight of the individual food is shown inside the photographs.

The foods were depicted in different portion sizes from 3 to 7 judged to be representative of the range of portion sizes actually consumed, placed on 2 different types of plates, flat and soup plates, common in the area. The portions were arranged in descending order with the biggest portion on the top. The name (in Spanish) and weight of the food is shown on the top of each photograph, the images were color prints in size 75 × 60 mm allowing eight photos to be displayed together on one A4 page. Figure 2 shows an example of photographs from the photo atlas. Additionally the photo atlas presents depicted raw ingredients (like tomatoes, onion, etc.) in different standardized sizes from 3 to 5 depending on the variety of actual sizes existing on the market, these photographs were useful when the subjects were describing the individual food items in mixed dishes such as soups, stews, etc.

### Photo kit

**Figure 3:**
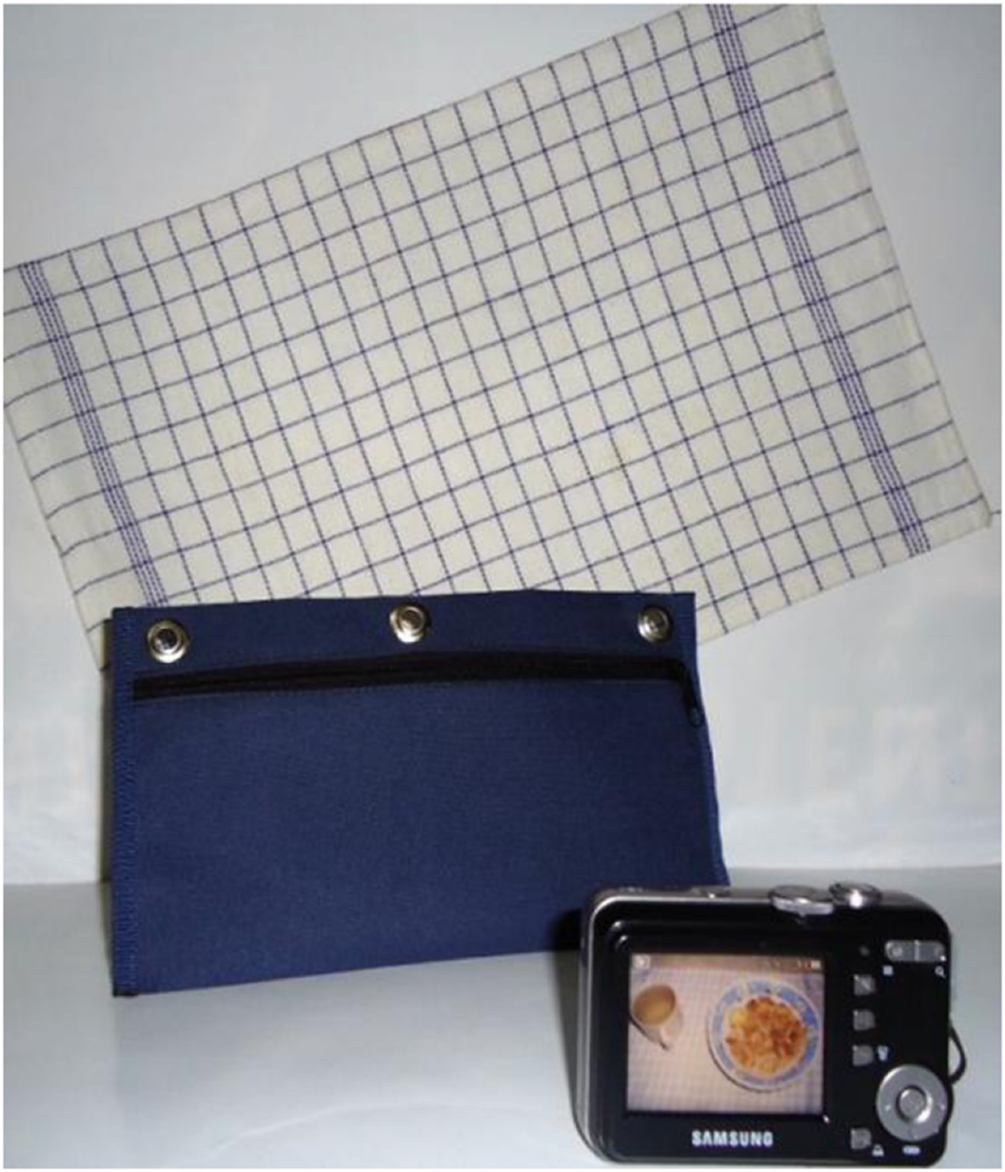
Photo kit: Digital camera, camera case and marked table mat.

A photo kit (Figure 3) to be used by the subjects for taking photographs of all their foods consumed during the test day was prepared, containing: a digital camera (Samsung Digimax S760, LCD screen 2.4 in) a camera case and a table mat. The table mat to put the plate on is marked with 1.5 cm grids providing a standard background, equal to that used in the photo atlas.

### Food photographs as a tool in 24-h dietary recall: FP 24-hR

The day before a test day a nurse, a nutritionist and an interviewer visited the women one by one in their homes and explained verbally the procedure of the study. When a woman voluntarily accepted to participate, she received verbal instructions, was given a demonstration and allowed to practice taking adequate photographs of her meals with easy-to-understand instructions.

**Figure 4:**
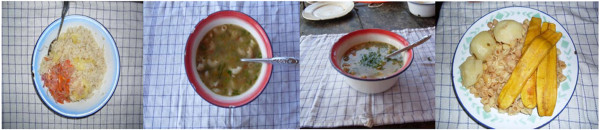
Representative photographs of breakfast, lunch (from two different angles) and dinner taken by a subject.

As a first step, the subjects took photographs of all their meals consumed during the test day with the following instructions: Place the plate with the food on the table mat, take two photographs before eating and two photographs after finishing if there are leftovers, one photograph at 90°, approximately 50 cm straight above the plate (hold the camera at a sufficient distance to see the whole marked table mat in the entire frame of camera screen and shoot, the size of the table mat was standardized to give ~50 cm distance in this position), and a second photograph with an approximate angle of 45° (take one step back from your original position fit the camera screen to cover the entire table mat and shoot). Both photographs are meant to span characteristics of appearance which are likely to influence perception of amounts from photographs, these characteristics are: area and height of pieces, mounds on a flat plate and depth in a soup plate, useful for a better estimation of the food portion sizes. Compliance with the method was good; 47 women were asked to participate, of which 45 (96%) accepted, they all took the photographs requested. Figure 4 shows the photographs taken by a subject during the test day showing breakfast, lunch (from two different angles) and dinner.

On the following day, as a second step, a trained interviewer (not the assistant who kept the weighed food record) asked the subject to recall the exact food intake during the preceding day, according to a four-stage, multiple-pass interviewing technique [1].

The multiple-pass 24-hr recall was conducted as described in Gibson, ( [2005]) with small modifications in the third pass, to estimate the amount of food and beverages consumed; during this pass, the subjects are to be asked to estimate the amount of food consumed; comparing the digital photographs they took on the test day with photographs of standard portion size in the photo atlas. At the same time the interviewer is to make her/his own comparison of the photographs and ascertain or correct the portion size selected. The subjects are also to be asked to describe some hidden foods which are not visible in the digital photographs.

### Reference method: WFR

The WFR, was run in parallel with the FP 24-hR. An assistant, who had previously been trained by a nutritionist, visited each subject during the preparation and consumption of her meals during the test day.

Before consumption of the meals, the amount of each food item and beverage was transferred to a clean dish, weighed (Ohaus Traveler TA 1501, capacity 1500 ± 0.1 g), and recorded separately, the same procedure was follow after consumption if there were leftovers, and the actual amount of each type of food eaten was subsequently calculated subtracting leftovers. In the case of mixed meals such as soups or stews, raw ingredients used in their preparation, were weighed (±0.1 g) and recorded individually, the final total weight of the mixed dish was weighed in the cooking pot, using a second scale with greater capacity (Ohaus Valor^TM^ 1000 V11P30, capacity 30 kg ± 5 g), also the individual served dish was weighed (±0.1 g) and recorded. The weight of each ingredient was calculated for individual consumption.

### Anthropometric measurements

Measurements of height and weight were performed by trained staff, using a digital electronic scale (Omron HBF-400), 150 kg ± 0.1 kg and a portable stadiometer ±1 mm. The subjects were lightly dressed and without shoes, when the measurements were taken, body mass index (BMI = weight [kg]/height [m^2^) was calculated and evaluated using the World Health Organization classification [25,26].

### Food intake and Nutrient calculation

A data base for nutrient calculation was elaborated in an excel file for most items with data from USDA National Nutrient Data Base for standard reference [27]. For a few items the Bolivian Food Composition Table was consulted [28]. The elaborated database contains 141 food items properly encoded. We chose to use the USDA reference database due to a lack of information in the Bolivian table about cooked food.

The data of food intake of the subjects was extracted from questionnaires (FP 24-hR) and records (WFR) of the 45 subjects who participated in the validation. The data were divided into 10 categories of food for comparing weighed and estimated amounts. The selected food categories reflect the composition of the diet pattern in this population as well as representing the source of certain nutrients of interest. The bread, rice and noodles category represents the staple cereal-based food. Potatoes and cassava are tubers mainly consumed in the area. Eggs and meat represent the main protein sources of their diet. Vegetables category was divided into leafy vegetables (spinach, lettuce, etc.) and vegetables (tomatoes, carrots, etc.), because leafy vegetables may be more difficult to estimate due to the volume they occupy does not represent their actual weight. And finally the category of beverages was added to evaluate the estimation of liquids.

All dietary information from WFR and FP 24-hR was coded according to the food code in the database. Food codes and amounts were entered into the excel files in order to compute the total amount consumed per day and the average daily energy and nutrient intake. The method has been validated with respect to actual intake of energy, protein, total fat, carbohydrates, dietary fiber, calcium, iron, zinc, selenium, folate, thiamin, niacin, β-carotenoids, and vitamins C, A and E. The macronutrients and fiber were selected because they are commonly requested in diet studies. The minerals and vitamins were selected according to their relevance to elucidate deficiencies present especially among rural populations in developing countries, and according to their different sources (i.e. folate, vitamins C, are mainly in vegetables; thiamin, niacin are mainly in cereal products, etc.).

### Statistical analysis

Normality of distribution of data was assessed by the Kolmogorov–Smirnov test and by visual inspection of histograms with reference to measures of skew and kurtosis. Logarithmic transformations were used, when appropriate, to normalize the data (food categories). The amounts of estimated food categories and calculated nutrient intake are reported at group level using medians and percentiles 25^th^, 75^th^ (for not normal distributed data) and means and standard errors (for normal distributed data).

To test the validity of the FP 24-hR, the mean or median difference in grams and percent of the intake between mean amounts actually eaten (WFR), and mean amounts estimated (FP 24-hR) were calculated and expressed at the category level. A negative difference is considered to indicate an underestimation of the weighed serving. The differences between amounts in portion sizes of food categories weighed and estimated were tested using Wilcoxon signed rank test (not normal distributed data) and differences between nutrient intakes estimated by FP 24-hR and WFR were tested using paired *t*-test (normal distributed data).

Pearson’s or Spearman’s rank correlation coefficients were calculated to assess the association between the weighed and estimated amount of food and between nutrient intakes assessed by both methods.

Agreement between both methods was assessed using the Bland-Altman regression; the mean differences of food amounts and nutrient intakes between both methods were plotted against its average value, and the 95% limits of agreement were marked. This kind of plot shows the magnitude of disagreement, allows outliers to be spotted and any trends to be identified; desirable agreement between the two methods would result in a difference of zero.

For all statistical tests the significance level was set up at P < 0.05; and the tests were carried out using SPSS version 18.0 (SPSS Inc., IBM corporation 2010, http://www.spss.com).

## Results

All the 45 subjects (100%) successfully completed the FP 24-hR. As 11 women had one of their meals (mid-afternoon snack or dinner) outside their home, complete data of WRF was available for 34 women (76%). The comparisons of food amounts estimated vs. weighed were made with the mean portions for each type of food from meals consumed at home for all 45 subjects. Comparison of nutrient intake calculated by both methods was analyzed for 34 subjects.

The subjects’ characteristics are presented in Table 1; the women aged 20 to 52, mean BMI 24.82 kg/m^2^. Fifty six percent were in the range of normal BMI values, while some of the women were underweight (7%), overweight (26%) and obese (11%).

**Table 1:** Characteristics of subjects

	**Women (n= 43)**		
	**Mean**	**SD**	**% (n)**
Age [years]	35	8.6	
Height [cm]	155.55	6.84	
Weight [kg]	59.76	8.70	
BMI^a^ [kg/m^2^]	24.82	4.06	100 (43)
Underweight	18.40	0.12	7 (3)
Normal weight	22.80	1.64	56 (24)
Overweight (Pre-obese)	27.31	1.64	26 (11)
Overweight (Obese class1)	32.79	1.61	11 (5)

^a^BMI [kg/m^2^, body mass index, classification according WHO [24]; underweight (<18.5), normal weight (18.50-24.99), pre-obese (25.00-29.99), obese class I (30.00-34.99).

### Comparison of food categories estimated vs. weighed amount

The data of food groups were not normally distributed; therefore the accuracy of the FP 24-hR method is presented for the foods listed as median values and percentiles (25^th^, 75^th^) of the amounts estimated in the questionnaires and the corresponding information of weighed food amounts recorded by assistants with WFR. This comparison was done for 10 major food categories: bread (n = 26), rice (n = 43), noodles (n = 43), potatoes (n = 80), cassava (n = 19), meat (n = 48), egg (n = 15), vegetables (n = 198), leafy vegetables (n = 17), and beverages (tea, milk or refreshments) (n = 19). The median amounts and percentiles (25^th^, 75^th^) of food estimated (FP 24-hR) and weighed (WFR) respectively are presented in Table 2 as well as the differences between the medians (in grams and percentage, respectively), and the percentiles of the differences are shown.

**Table 2:** Amount of food estimated by FP 24-hR and compared with amount weighed in WFR

**FOOD CATEGORY**	**(n)**	**FP 24-hR**	**WR**	** Sperman*r***	**Median difference FP24hR – WFR**^**a**^		**Bland Altman Analysis FP24hR – WFR (antilog)**^**b**^		
		**Median (P25, P75)**	**Median (P25, P75)**		**Median (P25, P75)**	**%**	**Geometric mean ratio**	**95% Limits of agreement**	
Bread [g]	26	55 (50, 60)	55 (47, 65)	0.81	-1.5 (-4.3,3.0)	-2.43	0.98	0.79	1.22
Rice [g]	43	165 (105, 200)	165 (108, 237)	0.95	-13.0 (-30.0, 5.0)	-6.76	0.93	0.71	1.20
Noodles [g]	43	175 (154, 256)	187 (150, 263)	0.93	-12.0 (-20.0, 9.0)	-5.41	0.97	0.73	1.28
Potatoes [g]	80	114 (61, 160)	115 (71, 168)	0.98	-5.0 (-10.0, 7.8)	-5.80	0.96	0.79	1.18
Cassava [g]	19	117 (64, 156)	108 (66, 143)	0.98	-1.0 (-8.0, 8.0)	-2.33	0.99	0.80	1.22
Meat [g]	48	36 (25, 51)	34 (26,49)	0.96	-2.0 (-4.0, 2.0)	-4.88	0.95	0.71	1.28
Egg [g]	15	50(50, 50)	54 (46, 57)	0.75	-3.0 (-7.0, -1.0)	-6.54	0.94	0.76	1.17
Vegetables [g]	198	25 (13, 43)	25 (14, 43)	0.96	-1.0 (-4.0, 2.0)	-5.44	0.98	0.65	1.43
Leafy vegetables [g]	17	25 (25, 50)	27 (20, 46)	0.90	2.0 (-5.0, 7.0)	8.70	1.09	0.70	1.69
Beverages [g]	19	250 (200, 325)	260 (210, 310)	0.95	4.0 (-10.0, 10.0)	1.63	1.01	0.93	1.10

^a^ Median difference between FP 24-hR and WFR, in grams with 25^th^, 75^th^ percentiles for the difference of each food category, and median of percentage percentage of the difference in parenthesis (calculated as: % of the difference = ((amount from FP 24hR- amount from WFR) / mean amount from WFR)*100).

^b^ Bland Altman analysis, show the antilog values of the mean difference between FP 24-hR and WFR, represented as the geometric mean ratio of amounts estimated by the FP 24-hR and weighed by WFR, and the 95% limits of agreement, represent the range of proportional agreement between both methods.

Most of the food categories were underestimated (ranging from −2.3% for cassava to −6.8% for rice), excepting for beverages (+1.6%) and leafy vegetables (+8.7%) which were somewhat overestimated. Data were analyzed with non-parametric tests; Wilcoxon signed rank test showed that the differences between estimated and weighed food are not significant (P > 0.05) except for rice (<0.001), potatoes (0.032), egg (0.030) and vegetables (0.039). Spearman^′^s correlations were calculated to determine the association at the individual level between the estimated amount and the actual weighed amount; all the food categories present a significant high correlation (*r* values from 0.75 for egg to 0.98 for potatoes and cassava).

**Figure 5:**
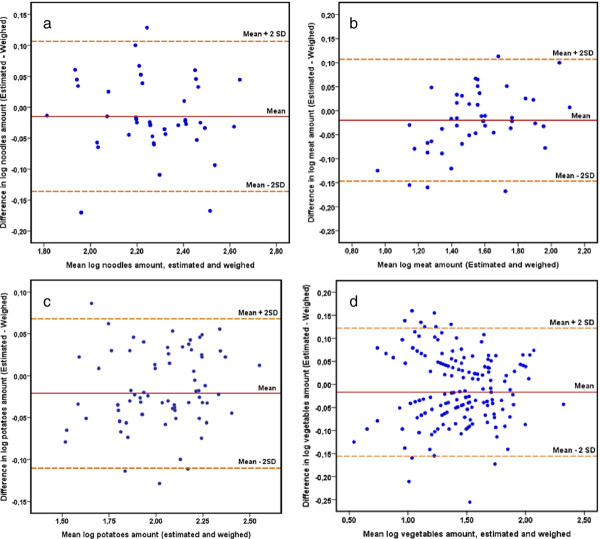
**Bland Altman plots for estimated and weighed food amount.** Differences between the log amounts of food portions estimated and weighed against their mean values, the solid line represents the average difference between the log estimated and the log weighed food amount; the dotted lines show the 95% log limits of agreement which, when calculating the antilog, represent the range of proportional agreement between both methods. **a)** Plot for noodles amount, **b)** Plot for potatoes, **c)** Plot for meat and **d)** Plot for vegetables. Plots show not systematic bias and that the range of proportional agreement is narrow enough to be confident using the photo method.

The agreement between the estimated and weighed amount was assessed by Bland Altman analysis of the log-transformed data, because they were not normally distributed, as shown in Figure 5 (for meat, noodles, potatoes and vegetables). The plots for the differences of food amounts, estimated (FP 24-hR) and weighed (WFR), show that most of the differences are between the limits of agreement at mean ± 2 SD, showing only a few outliers (from 0% for leafy vegetables and beverages to 8.3% for meat).

For all the food categories the results from Bland Altman analysis were back-transformed and are presented in Table 2, showing the geometric mean ratio of values by estimated and weighed food amount and the 95% limits of agreement. The geometric mean ratios are close to 1 and limits of agreement are narrow for most of the food categories. For beverages the geometric ratio is 1.01 and narrow limits of agreement (0.93 to 1.10), for leafy vegetables the geometric ratio is 0.98 with relatively broad limits of agreement (0.65 to 1.43).

### Comparison of nutrient intake calculated from FP 24-hR and WFR

The mean amount of nutrient intake from food consumption assessed by FP 24-hR and WFR respectively were calculated for energy, protein, total fat, carbohydrates, dietary fiber, calcium, iron, zinc, selenium, thiamin, niacin, folate, β-carotenoids, and vitamins C, A and E .

The data follow normal distribution and thus parametric tests were used for the analysis. The results of mean nutrient intake and standard errors are shown in Table 3 as well as the differences between means (in the corresponding units for each nutrient and in percentage) are presented. The differences are in the range of −0.90% (for Vitamin C) and −5.98% (for total fat), indicating that both methods are comparable, with small differences. All nutrient intakes were somewhat underestimated using the FP 24-hR method. Even though most of the differences are small they are statistically significant (paired *t*-test P < 0.05) except for calcium (P = 0.098), vitamin C (P = 0.528), vitamin A (P = 0.218) and β-carotenoids (P = 0.565).

**Table 3:** Mean nutrient intake and comparison of the results obtained with the methods: FP 24-hR and WFR

**NUTRIENT**	**FP 24-hR**		**WFR**		**Pearson*r***	**Mean difference FP24hR – WFR**^**a**^	**95% Limits of agreement**^**b**^	
	**Mean**	**SEM**	**Mean**	**SEM**				
Energy [kJ]	5854	262	6092	261	0.99	-238 (-3.99)	-683 (-11.5)	206 (3.5)
Protein [g]	46.70	2.23	48.95	2.33	0.99	-2.25 (-4.66)	-6.93 (-14.5)	2.43 (5.1)
Total fat [g]	23.59	1.12	25.09	1.12	0.96	-1.50 (-6.0)	-5.34 (-21.9)	2.34 (9.6)
Carbohydrate [g]	251	14	260	14	0.99	-8.45 (-3.2)	-32.66 (-12.8)	15.8 (6.2)
Dietary fiber [g]	15.6	1.0	16.2	1.1	0.99	-0.63 (-3.7)	-2.5 (-15.8)	1.6 (7.9)
Calcium [mg]	254	26	260	25	0.99	-6.20 (-2.4)	-48.1 (-18.7)	35.7 (13.9)
Iron [mg]	11.22	0.47	11.82	0.44	0.97	-0.60 (-5.1)	-1.92 (-16.7)	0.72 (6.3)
Zinc [mg]	6.54	0.30	6.91	0.30	0.98	-0.37 (-5.4)	-1.13 (-16.8)	0.38 (5.7)
Selenium [μg]	89.2	6.2	92.8	6.7	0.98	-3.55 (-3.8)	-19.2 (-21.1)	12.1 (13.3)
Vitamin C [mg]	65.1	7.1	65.7	7.25	0.99	-0.59 (-0.9)	-11.4 (-17.4)	10.2 (15.6)
Thiamin [mg]	0.78	0.05	0.81	0.05	0.98	-0.03 (-4.0)	-0.14 (-17.6)	0.08 (10.1)
Niacin [μg]	11.98	0.55	12.58	0.57	0.97	-0.60 (-4.7)	-2.15 (-17.5)	0.96 (7.8)
Folate total [μg]	177	13	185	13	0.98	-8.44 (-4.6)	-37.5 (-20.7)	20.6(11.4)
β-Carotenoids [μg]	3087	428	3126	462	0.99	-39.6 (-1.3)	-804 (-25.9)	787 (25.4)
Vitamin A [μg RE]	378	43	387	39	0.99	-8.31 (-2.1)	-85.4 (-22.3)	68.8 (18.0)
Vitamin E [mg]	2.44	0.15	2.50	0.14	0.97	-0.13 (-5.4)	-0.56 (-22.9)	0.29 (11.8)

^a^ Mean difference between FP 24-hR and WFR, expressed in the corresponding units for each nutrient and percentage in parenthesis. The percentage was calculated as: % of the mean difference = ((mean nutrient from FP 24hR- mean nutrient from WFR) / mean nutrient from FWR)*100.

^b^ 95% limits of agreement for the difference between the FP 24-hR and WFR, in the corresponding units for each nutrient and percentage in parenthesis, show the range of under and over-estimation for the agreement between both methods.

Significant correlation coefficients (Pearson) between all nutrient intakes estimated by the FP 24-hR and the WFR were obtained (*r* value from 0.96 to 0.99) indicating good association between both methods for all the nutrients.

**Figure 6:**
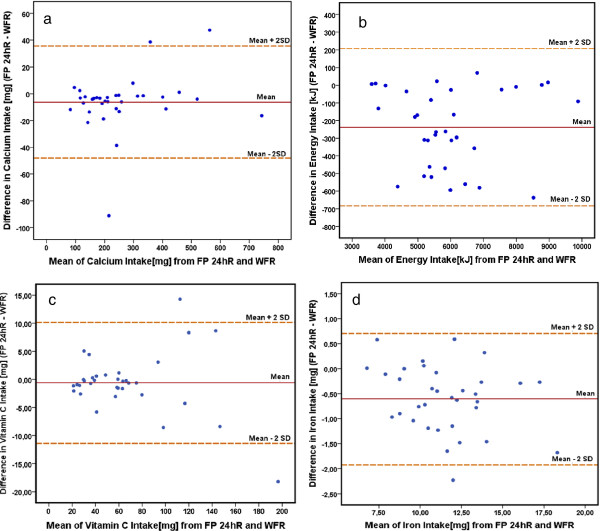
**Bland Altman plots for nutrient intakes calculated from FP 24-hR and WFR.** Differences between the mean dietary intakes of nutrients calculated from FP 24-hR and WFR against their mean values. The solid line represents the average difference between the FP 24-hR and WFR; the dotted lines show the 95% limits of agreement for the differences. **a)** Plot for energy intake, **b)** Plot for calcium intake, **c)** Plot for vitamin C intake and **d)** Plot for iron intake. The ranges of proportional agreement are narrow enough to be confident using the photo method.

In order to assess the agreement between the methods, for all nutrients, Bland Altman analysis was performed as shown in Figure 6 (for energy, calcium, vitamin C, and iron). Plots for each nutrient show a few outliers (from 0% for energy to 8.8% for calcium), the majority of the measurements were scattered along the equality line. The plots thus showed fairly good agreement between the two methods and also indicated that the differences (including the outliers) were random and did not exhibit any systematic bias.

In Table 3 the mean difference between the methods and 95% limits of agreement for the differences are presented in the corresponding units for each nutrient and in percentage; showing small differences from −0.90% (for vitamin C) to −5.98% (for total fat) and narrow limits of agreement for energy (−11.5 to 3.5%) and carbohydrates (−12.8 to 6.2%) and relatively broad but still acceptable limits are shown for β-carotenoids (−25.9 to 25.4%) and vitamin A (−22.9 to 11.8%).

## Discussion

The data analyses of individual food categories show that the FP 24-hR with digital photographs and a photo atlas was able to estimate the weights of food portion sizes adequately and gave results comparable to the actual consumed amounts recorded by the WFR. The modifications with digital photographs and a photo atlas added to the ability of the 24-h recall to minimize errors associated with the estimation of portion sizes, as well as the reduction of respondent burden. Therefore FP 24-hR represents a good alternative to the gold standard method (weighed food record) for estimating individual nutrient intakes, as it is demonstrated by the presented results.

Furthermore, recent studies show that the introduction of digital photographs taken by the subjects as a diet assessment method helps to estimate food intake and plate waste and this can reduce over and underestimates. This has been shown with children in cafeteria settings [17,20], with children at home [24,29], adults with intellectual disabilities living in the community [21], obese patients in hospital [22], as well as in college and university environments [16,18]. However, only a few studies have used digital photographs to estimate intake in free-living conditions [20] and with general populations [23]. Besides, to our knowledge this may be the first study using photographs to assess nutrient intake in rural populations in low-income countries.

Studies comparing food estimates from digital photographs and weighed records have found that the use of digital photographs results in small differences in the amount of food, in the range of −9.1 g to 18.3 g [16], and an underestimation of −6.6% for energy intake [20], although these differences are very small it has been reported that these underestimations were significantly different from the values obtained by the weighed record method. In the present study the comparison of the food amount evaluated by FP 24-hR and WFR has shown mean differences from −14.4 g to 4.5 g between the different food categories, where the differences were not statistically significant, excepting for some food categories (rice, potatoes, eggs, vegetables).

In the analysis of nutrient intake energy was underestimated by −3.92% and the underestimations are in the range of −0.90% for vitamin C to −5.98% for total fat. These underestimations were significant excepting for certain nutrients (calcium, vitamin A, C, and β-carotenoids). The significant differences found even when FP 24-hR and WFR have identical mean, may be due to the variance within each group owing to the high variability among individual food consumption (i.e. portion size of rice consumption varies from 44 g to 400 g between subjects), which is subsequently reflected in significant differences in nutrient intake. The small underestimation of most of the food groups and all the nutrients may be due to some hidden foods in the photographs making it difficult to estimate portion sizes, and failure in memory of the respondents to identify all the hidden foods in the photographs.

Notwithstanding the significant differences, the FP 24-hR showed high correlation coefficients in estimating portion sizes, in the range of 0.75 (for egg) to 0.98 (for potatoes and cassava), comparable to those reported in previous studies (>0.74) [12,15,19], where a photo atlas was used as a tool for quantifying portion size.

Moreover correlations between photographic food record and weighed dietary record, for energy intake, reported by previous authors were as high as: from 0.93 to 0.95 [20], 0.84 [30], from 0.44 to 0.48 [31], 0.73 [24], 0.79 [18] and 0.60 [32]. The correlation coefficient for energy intake reported in this study is 0.99. A few studies have reported correlation coefficients for macro and micronutrients; for protein 0.83, 0.48, and 0.61; for carbohydrates 0.55, 0.52 and 0.68; for fat 0.82, 0.46 and 0.50 were reported respectively by [24,31,32], the FP 24-hR found correlation of 0.99, 0.99 and 0.96 for protein, carbohydrates and fat respectively. Correlation for vitamins are reported in the range of 0.06 to 0.80 [31] and 0.30 to 0.86 [32], correlation for minerals from 0.34 to 0.57 [31], and from 0.21 to 0.74 [32], the present study reports correlations for vitamins and minerals in the range from 0.97 to 0.99.

The results found with the Bland Altman analysis showed that the majority of the measurements 95.2% for food categories and nutrient intake, were scattered along the mean difference line and close to the equality line (difference = 0). The plots thus show fairly good agreement between estimated and actual food consumed and indicate that the differences (including the outliers) were random and did not exhibit any systematic bias, being consistent over different levels of mean food amount. Results were similar to previous studies, which have reported that the bias between the use of digital photographs and weighed food records was consistent over different levels of energy intake, indicating that the two methods were comparable, and bias was very low [16,20].

In the analysis of food categories, the geometric mean ratios are close to 1 (from 0.93 for rice to 1.09 for leafy vegetables), and limits of agreement are narrow for most of the food categories. The ratios of proportional agreement indicate that for about 95% of the cases the estimated amounts will be between the values of the ratio respect to the weighed amount, for example for bread the geometric mean is 0.98 with limits of agreement from 0.79 to 1.22; thus FP 24-hR when is compared with WFR gives values by between 0.79 to 1.22 times the weighed amount of bread. The limits of agreement are relatively broad for vegetables (0.65 to 1.43) and leafy vegetables (0.70 to 1.69); this may be because the dispersion of the values in these two food categories increases as the weight increases.

The analysis of nutrient intake showed that the mean differences between FP 24-hR and WFR were low and the limits of agreement acceptable, for example the average energy intake estimated by the FP 24-hR was 5854 KJ, the mean difference when it was compared to WFR was −3.92% and the limits of agreement were from an under-estimate of −11.5% to an over-estimate of 3.5%, most of the nutrients showed similar narrow limits of agreement. The widest limits of agreement resulted for the intake of β-carotenoids which presented a small mean difference −1.27%, but the wide limits of agreement from an under-estimate of −25.9% to an over-estimate of 25.4%, similar for vitamin A. In spite of this, the limits are in an acceptable range to guarantee that the FP 24-hR can be used in place of the WFR for all the nutrients presented.

The small differences, high correlations and good agreement of the FP 24-hR with the WFR, may be because the food patterns in the study area are simple and less diversified than in urban populations where the food availability is wider and includes more processed food ready-to-eat, which might be more complicated to evaluate, in addition the use of digital photos and a 24-h recall questionnaire carried out by an interviewer make possible for the respondents to describe the hidden foods in the photographs or describe poor quality photographs, thus obtaining the most complete data possible. At the same time the volunteers were motivated with the FP 24-hR which involves the use of a simple but interesting and new device like a digital camera, because rural populations in developing countries are not so familiar with digital cameras. Another important factor that could enhance compliance with the method is that it is simple and fast, demands less than 2 minutes to take two pictures of each meal, which implies a maximum investment of 10 minutes per day to take digital photos of food consumption.

Very limited data are available about food and nutrient intake in rural areas in Bolivia. In this study we found an apparently low daily energy intake: mean 5.9 MJ, from 3.6 to 9.8 MJ in women 35 ± 8.6 years old. However, similar low energy intake for women in rural areas in South America has been reported previously, using different methods for measuring food consumption. In a study conducted in Calchaqui - Argentina, a 24-h recall and a semi-quantitative food-frequency questionnaire were applied and energy intake was estimated to be 6.6 MJ [33] in women 43 ± 15.2 years. Furthermore, in Ura Ayllu, Peru, low energy intake such as 5.3 to 7.5 MJ was reported by the weighed food record method in women 31 ± 6.3 years [34]. Also in a study using multiple pass 24-h recall in a Mexican population the energy intake was 5.9 MJ in women 32 ± 0.3 years [35].

The common food pattern in the currently studied population is based mainly on carbohydrates like: tubers (potatoes, cassava) and cereals (rice, bread, pasta); accompanied by small portions of protein from eggs or meat (mainly beef and chicken); oil or tallow as sources of fat, and a few vegetables and fruits. The composition of macronutrients as a percentage of total energy reflects the food pattern, in total carbohydrates 72 E%, protein 13 E%, and total fat 15 E%. The macronutrients consumption of the study group is within the dietary recommendation from the World Health Organization (Total carbohydrates 55–75 E%, protein 10–15 E%, and total fat 15–30%) [36]. However, the carbohydrates intake is nearly in the upper limit and the fat intake is nearly in the lower limit.

Despite the lower energy intake, 56% of the women had normal BMI (22.80 ± 1.64), 26% and 11% respectively were overweight or obese, and only 7% were underweight. These results are comparable to those found in rural areas with low energy intake such as in an study in Calchaqui- Argentina, which reported 39% of the women presenting normal weight [33].

A possible limitation in this study might be the undiversified food patterns of the population under study; the photo atlas was designed and developed in accordance of the specific food patterns in the area, as the method is aimed to be used in further studies of dietary assessment in the same area, another limitation is the relatively small number of the volunteers.

On the other hand the strengths of the study are: it was performed under the normal living conditions without disruption of the eating behavior, therefore the food consumed was representative of their habitual diet, and the inclusion of a digital camera which is a simple but interesting device for rural populations in developing countries may enhance the compliance with the method, and it may be used equally by both genders.

## Conclusions

Assessing the dietary intake in rural communities in developing countries is more complicated because the individuals are often illiterate, and not able to keep their own food records or use scales in a proper way in order to weigh consumed food. Other obstacles may be that they are busy working on farms, which leads to less spare time over for carrying out demanding dietary assessment methods or self-report methods. Besides it is well known that when keeping a weighed food diary there is always a risk that the subject will alter his normal diet, while with the interview method it is easier for the subject to make an incorrect statement about his food habits together with the difficulties in correct portion sizes estimation [10].

Thus, in order to reduce some of these drawbacks of the traditional methods used to assess the diet in rural populations, a FP 24-hR method is proposed and described, incorporating digital photographs taken by the subjects. This procedure is easier, faster, and less expensive to use than the WFR method, and it is less invasive; thus compliance may be enhanced. Furthermore the incorporation of a photo atlas facilitates and improves the important task of estimating portion sizes.

The validity of the method was assessed by several parameters. Firstly, the median and mean values obtained by the FP 24-hR compared well with those obtained by the WFR. Secondly, the Pearson and Spearman analysis showed high values of correlation coefficients, indicating good association between the two methods. Thirdly, the 95% limits of agreement showed acceptable values for the difference and, finally, Bland–Altman plots ensured the absence of systematic bias.

The FP 24-hR is associated and in agreement with the WFR. The photographs are useful as memory aids for the volunteers during 24-h recall and as an estimation tool for the interviewer. The proposed method is suitable for assessing the dietary intake of rural populations in low-income countries, and it may have important implications in clinical practice and research, representing a useful alternative to obtain accurate estimation of nutrient intakes.

## Abbreviations

FP 24-hR: Food photography 24-hour recall method; WFR: Weighed food record; BMI: Body mass index; SD: Standard deviation; SEM: Standard error; USDA: United States Department of Agriculture; WHO: World Health Organization.

## Competing interests

The authors declare that they have no competing interests.

## Authors’ contributions

**CEL** participated in the study design, as well as design of the questionnaires and photo atlas, performed data collection, statistical analyses and wrote the manuscript. **MEE** contributed in the design of the questionnaires, development of the photo atlas and data collection. **CA** contributed to the development of the photo atlas and data collection. **YG** participated in the study design, design of the questionnaires, statistical analyses, and manuscript revision. All authors read and approved the final manuscript.

## References

[B1] Gibson R Principles of Nutritional Assessment 2005 2 New York: Oxford University Press

[B2] Abebe Y Bogale A Hambidge KM Stoecker BJ Arbide I Teshome A Krebs NF Westcott JE Bailey KB Gibson RS Inadequate intakes of dietary zinc among pregnant women from subsistence households in Sidama, Southern Ethiopia Public Health Nutr 2008 11 04 379 386 17610755 10.1017/S1368980007000389

[B3] Rao S Kanade A Joshi S Yajnik C Community-specific modifications are essential for objective assessment of maternal dietary intake? Pune Maternal Nutrition Study Public Health Nutr 2009 12 09 1470 1476 10.1017/S1368980008004424 19105869

[B4] INE Instituto Nacional de Estadística: Bolivia Características de la Población. Censo Nacional de Población y Vivienda 2001, serie I Resultados Nacionales 2002 vol. 4 Document in Spanish

[B5] Steyn NP Nel JH Parker W-A Ayah R Mbithe D Dietary, social, and environmental determinants of obesity in Kenyan women Scandinavian J Public Health 2011 39 1 88 97 10.1177/1403494810384426 20851847

[B6] Gibson R Ferguson E An Iteractive 24-h recall for assessing the adequacy of iron and zinc intakes in developing countries 2008 In. Washington: Harvest Plus Technical Monograph Series

[B7] Gibson R Huddle J Suboptimal zinc status in pregnant Malawian women: its association with low intakes of poorly available zinc, frequent reproductive cycling, and malaria The Am J Clin Nutr 1998 67 4 702 709 10.1093/ajcn/67.4.702 9537617

[B8] Alemayehu AA Abebe Y Gibson RS A 24-h recall does not provide a valid estimate of absolute nutrient intakes for rural women in southern Ethiopia Nutrition 2011 27 9 919 924 10.1016/j.nut.2010.10.015 21295444

[B9] Poslusna K Ruprich J de Vries JHM Jakubikova M van't Veer P Misreporting of energy and micronutrient intake estimated by food records and 24 hour recalls, control and adjustment methods in practice Br J Nutr 2009 101 SupplementS2 S73 S85 19594967 10.1017/S0007114509990602

[B10] Bingham SA Limitations of the Various Methods for Collecting Dietary Intake Data Ann Nutr Metab 1991 35 3 117 127 10.1159/000177635 1952811

[B11] Chambers Iv E Godwin SL Vecchio FA Cognitive strategies for reporting portion sizes using dietary recall procedures J Am Diet Assoc 2000 100 8 891 897 10.1016/S0002-8223(00)00259-5 10955046

[B12] Nelson M Atkinson M Darbyshire S Food Photography I: the perception of food portion size from photographs Br J Nutr 1994 72 05 649 663 10.1079/BJN19940069 7826990

[B13] Nelson M Atkinson M Darbyshire S Food photography II: use of food photographs for estimating portion size and the nutrient content of meals Br J Nutr 1996 76 01 31 49 10.1079/BJN19960007 8774215

[B14] Ovaskainen ML Paturi M Reinivuo H Hannila ML Sinkko H Lehtisalo J Pynnonen-Polari O Mannisto S Accuracy in the estimation of food servings against the portions in food photographs Eur J Clin Nutr 2007 62 5 674 681 17440523 10.1038/sj.ejcn.1602758

[B15] Turconi G Guarcello M Berzolari FG Carolei A Bazzano R Roggi C An evaluation of a colour food photography atlas as a tool for quantifying food portion size in epidemiological dietary surveys Eur J Clin Nutr 2005 59 8 923 931 10.1038/sj.ejcn.1602162 15928683

[B16] Williamson DA Allen HR Martin PD Alfonso AJ Gerald B Hunt A Comparison of digital photography to weighed and visual estimation of portion sizes J Am Diet Assoc 2003 103 9 1139 1145 10.1016/S0002-8223(03)00974-X 12963941

[B17] Swanson M Digital Photography as a Tool to Measure School Cafeteria Consumption J Sch Health 2008 78 8 432 437 10.1111/j.1746-1561.2008.00326.x 18651930

[B18] Wang DH Kogashiwa M Ohta S Kira S Validity and reliability of a dietary assessment method: the application of a digital camera with a mobile phone card attachment JNSV 2002 48 6 498 504 10.3177/jnsv.48.498 12775117

[B19] Robson PJ Livingstone MBE An evaluation of food photographs as a tool for quantifying food and nutrient intakes Public Health Nutr 2000 3 02 183 192 10948385 10.1017/s1368980000000215

[B20] Martin CK Han H Coulon SM Allen HR Champagne CM Anton SD A novel method to remotely measure food intake of free-living individuals in real time: the remote food photography method Br J Nutr 2009 101 03 446 456 10.1017/S0007114508027438 18616837 PMC2626133

[B21] Humphries K Traci MA Seekins T Food on Film: Pilot Test of an Innovative Method for Recording Food Intake of Adults with Intellectual Disabilities Living in the Community J Appl Res Intellectual Disabil 2008 21 2 168 173 10.1111/j.1468-3148.2007.00392.x

[B22] Gregory R Walwyn L Bloor S Amin S A feasibility study of the use of photographic food diaries in the management of obesity Practical Diabetes International 2006 23 2 66 68 10.1002/pdi.899

[B23] Kikunaga S Tin T Ishibashi G Wang D-H Kira S The application of a handheld personal digital assistant with camera and mobile phone card (Wellnavi) to the general population in a dietary survey J Nutr Sci Vitaminol 2007 53 2 109 116 10.3177/jnsv.53.109 17615997

[B24] Small L Sidora-Arcoleo K Vaughan L Creed-Capsel J Chung K-Y Stevens C Validity and reliability of photographic diet diaries for assessing dietary intake among young children ICAN: Infant, Child, & Adolescent Nutrition 2009 1 1 27 36 10.1177/1941406408330360

[B25] WHO, World Health Organization Use and interpretation of anthropometric indicators of nutritional status Bulletin of the World Health Organization 1986 929 941 http://whqlibdoc.who.int/bulletin/1986/Vol64-No6/bulletin_1986_64(6)_929-941.pdf PMC2490974 3493862

[B26] Frisancho A Anthropometric standards for the assessment of growth and nutritional status 1990 Ann Arbor: The University of Michigan Press

[B27] USDA National Nutrient Database for Standard Reference http://www.nal.usda.gov/fnic/foodcomp/search

[B28] INLASA Tabla Boliviana de Composicion de Alimentos 2005 La Paz: Ministerio de Salud y Deportes Document in Spanish

[B29] Matthiessen TB Steinberg FM Kaiser LL Convergent validity of a digital image-based food record to assess food group intake in youth J Am Diet Assoc 2011 111 5 756 761 10.1016/j.jada.2011.02.004 21515125

[B30] Suzuki AMM Hattori I Egami I Wakai K Tamakoshi A Ando M Nakayama T Ohno Y Kamura T Inter-observer agreement and validity of photographic dietary assessment Jpn Public Health 2002 49 749 758 12355869

[B31] Higgins JA LaSalle AL Zhaoxing P Kasten MY Bing KN Ridzon SE Witten TL Validation of photographic food records in children: are pictures really worth a thousand words[quest] Eur J Clin Nutr 2009 63 8 1025 1033 10.1038/ejcn.2009.12 19259111

[B32] Wang D-H Kogashiwa M Kira S Development of a New Instrument for Evaluating Individuals' Dietary Intakes Journal of the American Dietetic Association 2006 106 10 1588 1593 10.1016/j.jada.2006.07.004 17000191

[B33] Bassett MN Romaguera D Samman N Nutritional status and dietary habits of the population of the Calchaqui Valleys of Tucuman, Argentina Nutrition 2010 27 11–12 1130 1135 21890324 10.1016/j.nut.2010.12.016

[B34] Margaret AG “No somos iguales”: The effect of household economic standing on women's energy intake in the Andes Social Science & Medicine 2004 58 11 2291 2300 10.1016/j.socscimed.2003.08.018 15047085

[B35] Batis C Hernandez-Barrera L Barquera S Rivera JA Popkin BM Food Acculturation Drives Dietary Differences among Mexicans, Mexican Americans, and Non-Hispanic Whites The Journal of Nutrition 2011 141 10 1898 1906 10.3945/jn.111.141473 21880951 PMC3174859

[B36] WHO, World Health Organization Dietary Recommendations in the Report of a Joint WHO/FAO Expert Consultation on Diet, Nutrition and the Prevention of Chronic Diseases (WHO Technical Report Series 916) 2003 http://whqlibdoc.who.int/trs/WHO_TRS_916.pdf 12768890

